# Supplementary material for: TWIST1 and chromatin regulatory proteins interact to guide neural crest cell differentiation
Source: eLife. 2021 Feb 8;10:e62873. doi: 10.7554/eLife.62873 (PMC7968925; doi:10.7554/eLife.62873)
Supplement: Supplementary file 3. — Cell line of origin of the candidate is listed. Expression data of the embryonic head was from published study (Fan et al., 2016). PSM: peptide sequence matches. Log2 FC = log2 transformed PSM fold-change between TWIST1-BirA*HA and GFP transfected O9-1 cells. Adjusted p-value was computed from dataset from O9-1 cell line, generated by the likelihood ratio test corrected by the Benjamini and Hochberg method in EdgeR (Robinson et al., 2010). Diffusion Rank: The rank of candidates in heat diffusion from genes associated with human and mouse facial malformation. [file elife-62873-supp3.docx]

### Table S3. Information on BioID candidates selected for validation

Cell line of origin of the candidate is listed. Expression data of the embryonic head was from published study (Fan *et al.*, 2016). PSM: peptide sequence matches. Log2 FC = log2 transformed PSM fold-change between TWIST1-BirA*HA and GFP transfected O9-1 cells. Adjusted p-value was computed from dataset from O9-1 cell line, generated by the likelihood ratio test corrected by the Benjamini & Hochberg method in EdgeR (Robinson *et al.*, 2010). Diffusion Rank: The rank of candidates in heat diffusion from genes associated with human and mouse facial malformation.

| Gene ID | Function | Cell line/s | Expression in embryonic head | PSM | Log2FC | Adjusted  p-value | Diffusion Rank |
| --- | --- | --- | --- | --- | --- | --- | --- |
| Tfe3 | bHLH factor; TGF-beta signaling | O9-1 & 3T3 | Y | 12.3 | 1.639 | 0.001706 | 24 |
| Smarce1 | O9-1Chromatin regulator SWI/SNF complex | O9-1 | Y | 15.5 | 1.762 | 0.0001697 | 19 |
| Chd7 | Chromodomain Helicase DNA Binding Protein | O9-1 | Y | 11.4 | 2.212 | 0.0001491 | 16 |
| Chd8 | Chromodomain Helicase DNA Binding Protein | O9-1 | Y | 46.5 | 1.656 | 2.45E-09 | 28 |
| Prrx1 | DNA binding, transcriptional coactivation | O9-1 & 3T3 | Y | 25.1 | 3.55 | 1.05E-13 | 8 |
| Prrx2 | DNA binding, transcriptional coactivation | O9-1 & 3T3 | Y | 10.4 | 3.012 | 9.78E-06 | 7 |
| Dvl1 | WNT signaling receptor Dishevelled | O9-1 & 3T3 | Y | 3 | 2.949 | 0.02028 | 1 |
| Whsc1 | Histone methylation H2K36 | O9-1 | Y | 12.4 | 3.324 | 4.74E-07 | 14 |
| Tcf12 | bHLH factor | O9-1 & 3T3 | Y | 10.3 | 6.386 | 8.10E-09 | 8 |
| Tcf4 | bHLH Factor | O9-1 & 3T3 | Y | 6.4 | 5.7 | 4.58E-06 | 16 |
